# Profiling the miRNA-mRNA-lncRNA interaction network in MSC osteoblast differentiation induced by (+)-cholesten-3-one

**DOI:** 10.1186/s12864-018-5155-2

**Published:** 2018-10-29

**Authors:** Qiuke Hou, Yongquan Huang, Yamei Liu, Yiwen Luo, Bin Wang, Rudong Deng, Saixia Zhang, Fengbin Liu, Dongfeng Chen

**Affiliations:** 1 Department of Anatomy, The Research Centre of Integrative Medicine, Guangzhou University of Chinese Medicine, Guangzhou, 510000 Guangdong People’s Republic of China; 2 The First Affiliated Hospital of Guangzhou University of Chinese Medicine, Guangzhou, 510000 Guangdong People’s Republic of China; 3 Department of Orthopaedics, The Second Affiliated Hospital of Guangzhou University of Chinese Medicine, Guangzhou, 510000 Guangdong People’s Republic of China; 4 Department of Diagnosis of Traditional Chinese Medicine, Guangzhou University of Chinese Medicine, Guangzhou, 510000 Guangdong People’s Republic of China; 5 Department of Trauma, The Third Affiliated Hospital of Guangzhou University of Chinese Medicine, Guangzhou, 510000 Guangdong People’s Republic of China

**Keywords:** (+)-cholesten-3-one, Osteoblastic differentiation, Mesenchymal stem cells, miRNA-mRNA-lncRNA

## Abstract

**Background:**

Our previous study showed that (+)-cholesten-3-one (CN) has the potential to induce the osteoblastic differentiation of mesenchymal stem cells (MSCs). However, the roles of CN in targeting miRNA-mRNA-lncRNA interactions to regulate osteoblast differentiation remain poorly understood.

**Results:**

A total of 77 miRNAs (36 upregulated and 41 downregulated) and 295 lncRNAs (281 upregulated and 14 downregulated) were significantly differentially expressed during CN-induced MSC osteogenic differentiation. Bioinformatic analysis identified that several pathways may play vital roles in MSC osteogenic differentiation, such as the vitamin D receptor signalling, TNF signalling, PI3K-Akt signalling, calcium signalling, and mineral absorption pathways. Further bioinformatic analysis revealed 16 core genes, including 6 mRNAs (Vdr, Mgp, Fabp3, Fst, Cd38, and Col1a1), 5 miRNAs (miR-483, miR-298, miR-361, miR-92b and miR-155) and 5 lncRNAs (NR_046246.1, NR_046239.1, XR_086062.1, XR_145872.1 and XR_146737.1), that may play important roles in regulating the CN-induced osteogenic differentiation of MSCs. Verified by the luciferase reporter, AR-S, qRT-PCR and western blot assays, we identified one miRNA (miR-298) that may enhance the osteogenic differentiation potential of MSCs via the vitamin D receptor signalling pathway.

**Conclusions:**

This study revealed the global expression profile of miRNAs and lncRNAs involved in the Chinese medicine active ingredient CN-induced osteoblast differentiation of MSCs for the first time and provided a foundation for future investigations of miRNA-mRNA-lncRNA interaction networks to completely illuminate the regulatory role of CN in MSC osteoblast differentiation.

**Electronic supplementary material:**

The online version of this article (10.1186/s12864-018-5155-2) contains supplementary material, which is available to authorized users.

## Background

Mesenchymal stem cells (MSCs) are the most promising bone regeneration and repair cells due to their osteogenic potential [1]. Though it has great potential for clinical applications [2], the osteogenic differentiation of MSCs is precisely regulated and coordinated by mechanical and molecular signals from the extracellular environment and involves complex pathways at the transcriptional and post-transcriptional levels [3, 4] that remain largely unexplored. Steroids, especially (+)-cholesten-3-one (CN), have therapeutic potential to enhance the osteoblast differentiation of MSCs and increase the expression of osteogenesis-specific factors, as reported in our previous study [5, 6]. However, previous studies were focused on the transcriptional, post-transcriptional and epigenetic levels, and the exact molecular mechanisms involved in osteogenesis and pre-transcriptional levels require further study.

MicroRNAs (miRNAs) are a class of small-molecule (17–25 nucleotides), single-chain RNAs that do not encode any proteins and have been identified as regulators of biological processes [7, 8]. MiRNAs regulate gene expression mainly by binding to the 3′-untranslated regions (3’-UTRs) of target mRNAs, resulting in their degradation or inhibition of their translation [9, 10], and miRNAs can also regulate gene expression by binding to 5’-UTR [11, 12], even in coding regions. More recently, advances in high-throughput technologies and computational methods have enabled unprecedented analysis of long-chain noncoding RNAs (lncRNAs) (another class of noncoding RNAs) [13, 14]. Increasing evidence [15–17] has shown that lncRNAs regulate gene expression by interacting with DNA, RNA or proteins, but identifying the functions of individual lncRNAs remains challenging [18–20]. Previous research has focused on exploring how noncoding RNAs regulate mRNA expression; however, some scholars believe that miRNAs, lncRNAs and mRNA can form a well-regulated interaction network [21, 22]. Previous studies [21, 22] have reported that miRNA-mRNA-lncRNA interaction networks play important roles in biological processes. However, none of these interaction networks have been shown to regulate the CN-induced osteoblast differentiation of MSCs. Therefore, further research is required to investigate the miRNA-mRNA-lncRNA interaction network in the CN-induced osteoblast differentiation of MSCs.

Herein, microarray analysis was used to explore the differential expression of miRNAs and lncRNAs during CN-induced MSC osteoblast differentiation. Subsequently, bioinformatics analyses and in vitro experiments were conducted to explore the miRNA-mRNA-lncRNA interaction network during CN-induced MSC osteoblast differentiation. Our findings lay the basis for future investigations of the miRNA-mRNA-lncRNA interaction network in MSC osteogenesis regulated by CN.

## Results

### Differentially expressed miRNA and lncRNA profiles during CN-induced MSC osteoblastic differentiation

**Fig. 1:**
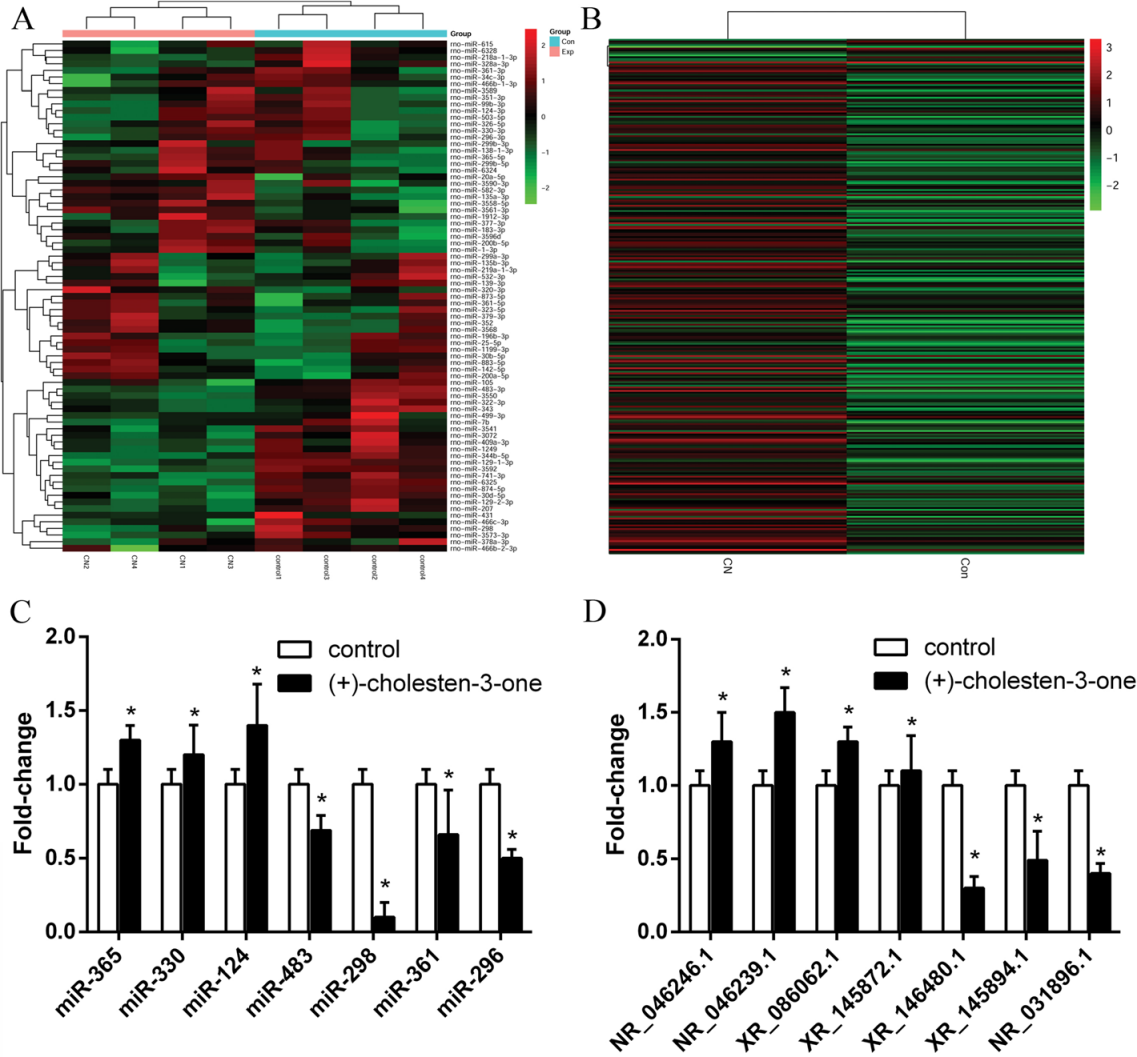
Heat map of miRNA and lncRNA differential expression during the CN-induced osteoblast differentiation of MSCs. **a** In total, 77 miRNAs were differentially expressed, including 36 that were upregulated and 41 that were downregulated. **b** In total, 295 lncRNAs were differentially expressed, including 281 that were upregulated and 14 that were downregulated. **c** qRT-PCR verification of the miRNA microarray results. Compared with the control group, the expression levels of miR-365, miR-330 and miR-124 were higher in CN-induced MSCs, and those of miR-483, miR-298, miR-361 and miR-296 were lower in CN-induced MSCs, which was consistent with the miRNA microarray results. **d** qRT-PCR verification of the lncRNA microarray results. Compared with the control group, the expression levels of NR_046246.1, NR_046239.1, XR_086062.1, and XR_145872.1 were higher in CN-induced MSCs, and those of NR_031896.1, XR_146480.1 and XR_145894.1 were lower in CN-induced MSCs, which was consistent with the lncRNA microarray results. *P* < 0.05, compared to the control group. The color scale shown on the top illustrates the relative expression level of the genes between two group: red denotes high expression levels, whereas green denotes low expression levels

A total of 77 differentially expressed miRNAs were identified during CN-induced osteoblast differentiation compared to uninduced MSCs (*P* < 0.05); among them, 36 were upregulated and 41 were downregulated. In addition, a total of 295 lncRNAs were differentially expressed after the CN-induced osteoblast differentiation of MSCs (*P* < 0.05), of which 281 were upregulated and 14 were downregulated (Fig. 1a & b; Additional file 1).

Seven miRNAs (miR-483, miR-298, miR-361, miR-296, miR-365, miR-330 and miR-124) and 7 lncRNAs (NR_046246.1, NR_046239.1, NR_031896.1, XR_086062.1, XR_146480.1, XR_145872.1 and XR_145894.1) were randomly selected to verify the reliability of the microarray data via qRT-PCR, which showed results similar to those of the microarray profiles (Fig. 1c & d).

### Candidate mRNA prediction and coding-noncoding gene co-expression (CNC) network construction during CN-induced MSC osteoblast differentiation

**Fig. 2:**
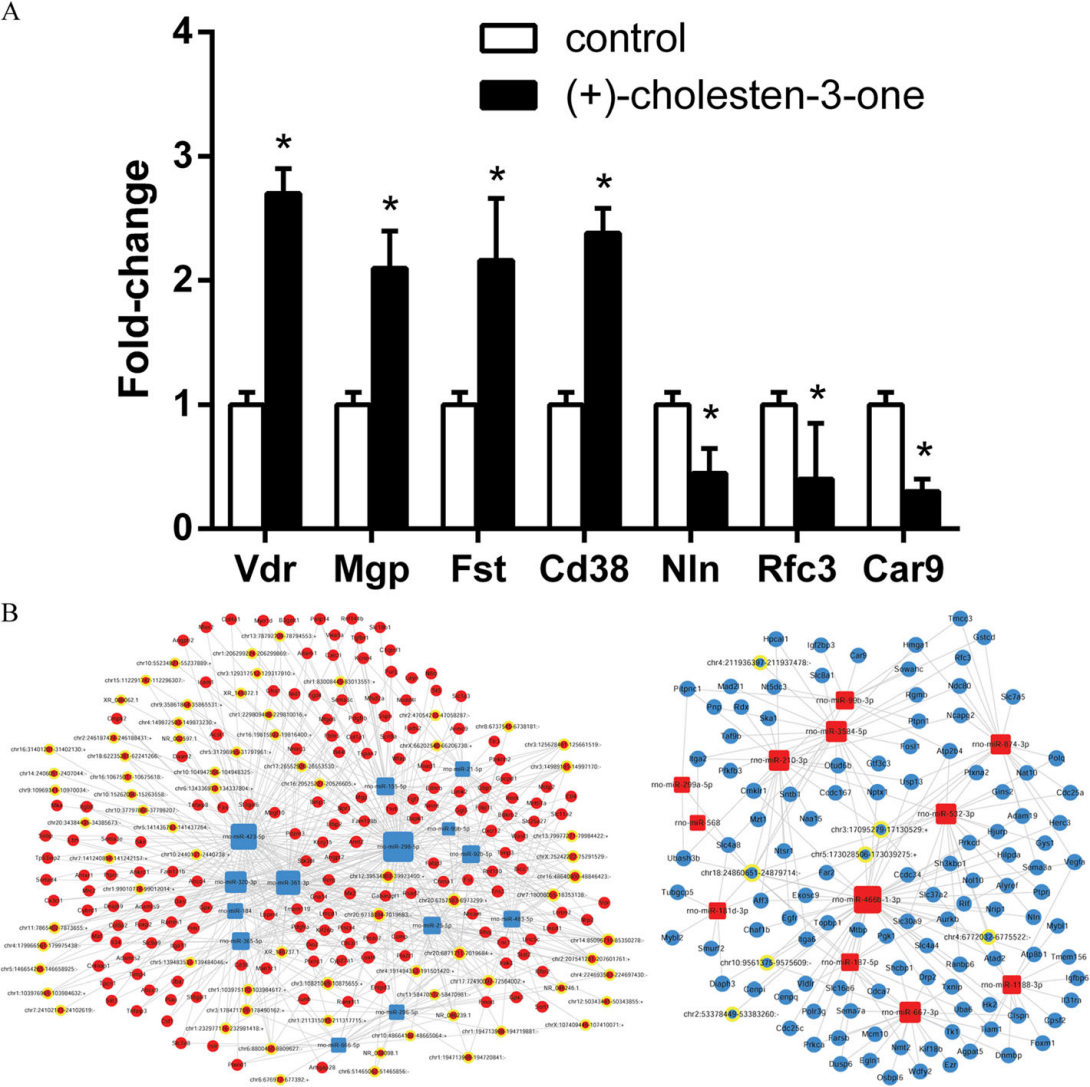
Candidate mRNA predictions and CNC network construction during CN-induced MSC osteoblast differentiation. **a** In total, 443 mRNAs were predicted via mRBase targets based on the miRNA and lncRNA microarray. Seven mRNAs (Vdr, Mgp, Fst, Cd38, Nln, Rfc3 and Car9) were selected for validation via qRT-PCR, and the results were consistent with the predicted target genes. **b** CNC networks during the CN-induced osteoblast differentiation of MSCs. The dots represent mRNAs, the dots in circles represent lncRNAs, and the squares represent miRNAs. All the red dots represent upregulation, and all the blue dots represents downregulation. *P* < 0.05, compared to the control group

Because the differential expression of mRNAs during CN-induced MSC osteoblast differentiation was not detectable in this study, mRNA predictions were made using miRanda (http://miranda.org.uk/) based on the miRNA and lncRNA microarray. A total of 443 possible mRNAs were predicted to targeted by differentially expressed miRNA and lncRNA during CN-induced osteoblast differentiation compared to uninduced MSCs (*P* < 0.05); of these, 223 were predicted to be upregulated, and 220 were predicted to be downregulated (*P* < 0.05) (Additional file 2). To confirm the reliability of the predicted target genes, 7 mRNAs (Vdr, Mgp, Fst, Cd38, Nln, Rfc3 and Car9) were selected for validation with qRT-PCR, which showed results similar to the predicted target genes (Fig. 2a).

We then constructed a CNC network to study the interactions between miRNAs, mRNAs and lncRNAs that were differentially expressed during CN-induced MSC osteoblast differentiation. The CNC network was based on normalized signal intensity and plotted using Cytoscape 3.0 (Fig. 2b). For visual analysis, the Pearson correlation coefficient was calculated first, and only the strongest correlations were included (Pearson correlation coefficient > 0.9). A node represented a gene, and the edges between two nodes represented the connection between two genes. Degrees were defined as the number of directly related neighbours. Then, the relationship among miRNAs-mRNAs-lncRNAs was integrated into the CNC network based on negative regulation. Finally, we identified 277 target mRNAs according to the degree values; of these, 165 were upregulated, and 112 were downregulated. Each mRNA corresponds to one or more miRNA and lncRNA and vice versa (Additional file 3).

### Bioinformatic analysis of candidate genes during CN-induced MSC osteoblast differentiation

**Fig. 3:**
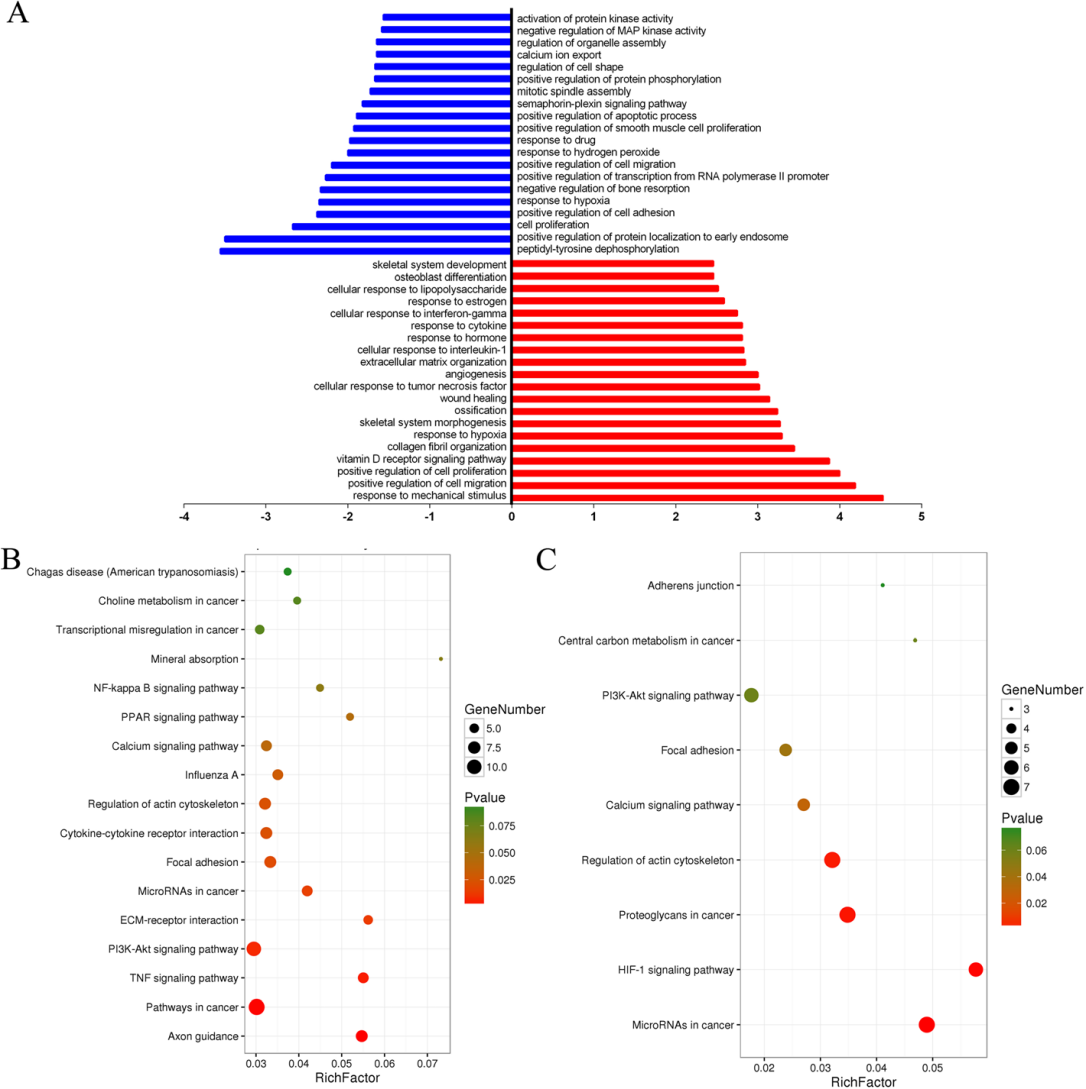
GO and KEGG analyses of predicted target genes during CN-induced MSC osteoblast differentiation. **a** GO analysis of 443 predicted target genes. In total, 174 biological processes were enriched; upregulation is shown in red, and downregulation is shown in blue. The y-axis shows the top ten biological processes, and the x-axis shows the negative logarithm of the *p* value (-LgP) of each biological process. **b** KEGG analysis of upregulated predicted target genes. **c** KEGG pathway analysis of downregulated predicted target genes. In both B & C, the y-axis shows the pathway category, and the x-axis shows the richness factor. A larger richness factor indicates greater enrichment. The size of the bubble indicates the number of genes in the pathway, and the colour of the bubble represents the range of *P* values

First, gene ontology (GO) cluster analysis was conducted to further explore the biological processes involving the 277 candidate mRNAs. As a result, 174 biological processes were enriched, including 113 that were upregulated and 61 that were downregulated (Additional file 4). Between the upregulated biological processes, the response to mechanical stimulus, positive regulation of cell migration, positive regulation of cell proliferation, vitamin D receptor signalling pathway, and collagen fibril organization processes may be related to CN-induced MSC osteoblast differentiation. Among the downregulated biological processes, the peptidyl-tyrosine dephosphorylation, positive regulation of protein localization to early endosome, cell proliferation, positive regulation of cell adhesion, and response to hypoxia processes may be related to CN-induced MSC osteoblast differentiation (Fig. 3a; Additional file 4). In addition, 59 upregulated genes and 45 downregulated genes were identified by GO cluster analysis.

Next, Kyoto Encyclopedia of Genes and Genomes(KEGG) pathway analysis was conducted to further explore the signalling pathways involving the 277 candidate mRNAs. In total, 26 significant signalling pathways related to CN-induced MSC osteogenic differentiation were enriched, including 17 upregulated pathways and 9 downregulated pathways (Additional file 5). Among the upregulated pathways, the axon guidance, TNF signalling, PI3K-Akt signalling, calcium signalling, and mineral absorption pathways may be related to CN-induced MSC osteoblast differentiation. Among the downregulated pathways, the microRNAs in cancer, HIF-1 signalling, proteoglycans in cancer, regulation of actin cytoskeleton, and focal adhesion pathways may be related to CN-induced MSC osteoblast differentiation (Fig. 3b & c; Additional file 5). In addition, 49 upregulated genes and 21 downregulated genes were identified by KEGG pathway analysis.

### Core CNC networks during CN-induced MSC osteoblast differentiation

**Fig. 4:**
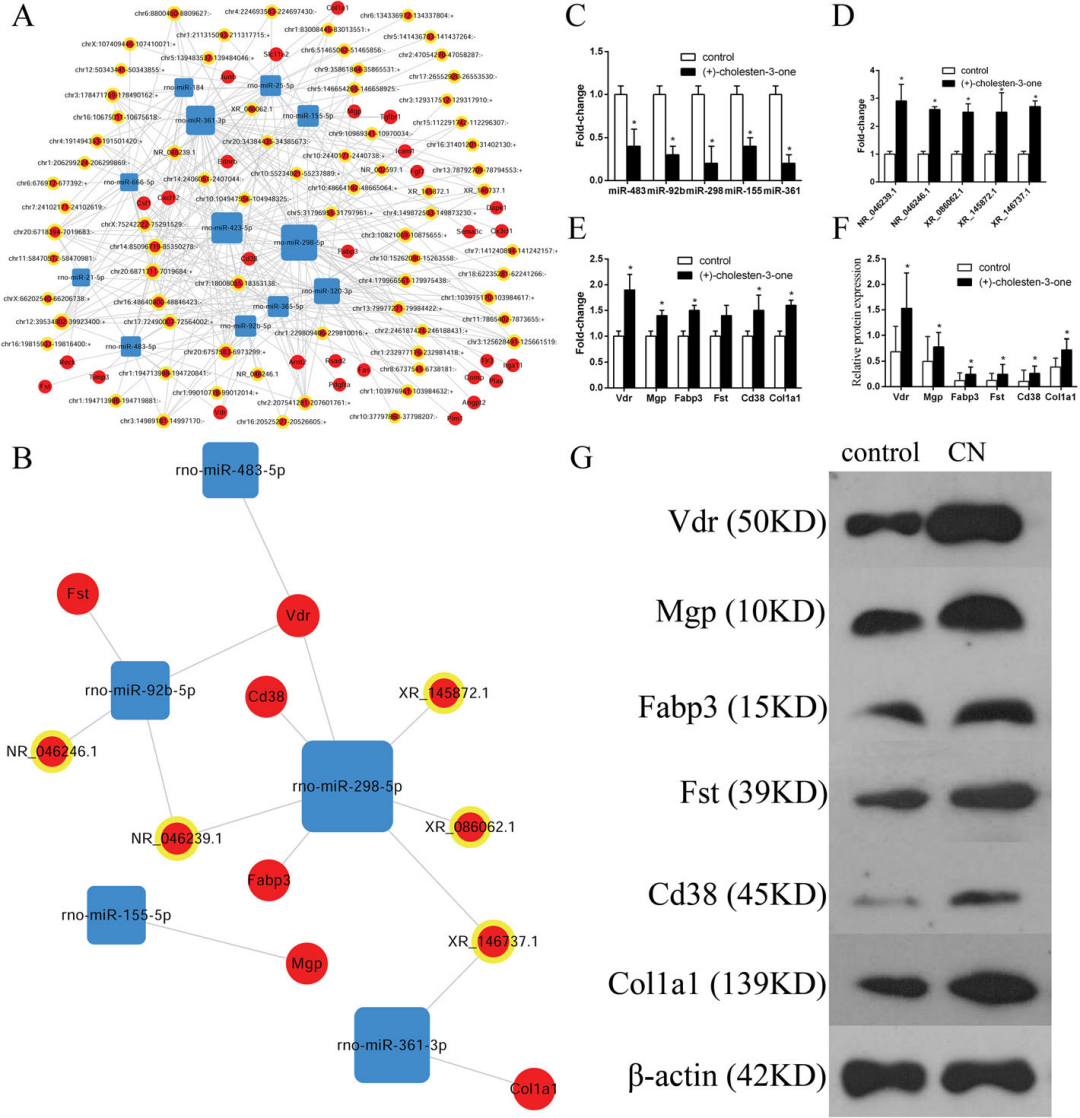
Core CNC networks during the CN-induced osteoblast differentiation of MSCs. **a** Core CNC network based on the 29 core genes obtained from GO cluster analysis and KEGG pathway analysis. **b** Sub-core CNC networks based on the 6 core genes obtained from online databases and core CNC networks. **c** qRT-PCR verification of miRNA expression. Compared with the control group, the expression levels of all the core miRNAs were lower in the CN-induced MSC group, which was consistent with the sub-core CNC network results. **d** qRT-PCR verification of lncRNA expression. Compared with the control group, the expression levels of all the core lncRNAs were higher in the CN-induced MSC group, which was consistent with the sub-core CNC network results. **e** qRT-PCR verification of mRNA expression. Compared with the control group, the expression levels of all the core mRNAs were higher in the CN-induced MSC group, which was consistent with the sub-core CNC network results. **f** and **g** Western blot verification of mRNA expression; the results were similar to the qRT-PCR results. *P* < 0.05, compared to the control group

The core CNC network were then constructed based on the core genes(29 mRNAs), which were obtained from the same genes from GO and KEGG analyses (Fig. 4a; Additional file 6). The osteogenesis-related genes were identified using online databases (https://geneticassociationdb.nih.gov/, https://www.ncbi.nlm.nih.gov/gene/) and our core CNC networks. At last 6 core genes (Vdr, Mgp, Fabp3, Fst, Cd38, and Col1a1) were identified, and 5 core miRNAs (miR-483, miR-298, miR-361, miR-92b and miR-155) and 5 core lncRNAs (NR_046246.1, NR_046239.1, XR_086062.1, XR_145872.1 and XR_146737.1) were correspondingly identified (Fig. 4b; Additional file 7).

To confirm the reliability of the sub-core CNC networks and the online databases, the 5 core miRNAs, 5 core lncRNAs and 6 core mRNAs were verified via qRT-PCR and western blot analysis. The qRT-PCR (Fig. 4c, d & e) and western blot (Fig. 4f & g) results confirmed that the expression levels of these 16 core genes were consistent with the sub-core CNC networks and online databases.

### miR-298 directly targets Vdr during the CN-induced osteoblast differentiation of MSCs

**Fig. 5:**
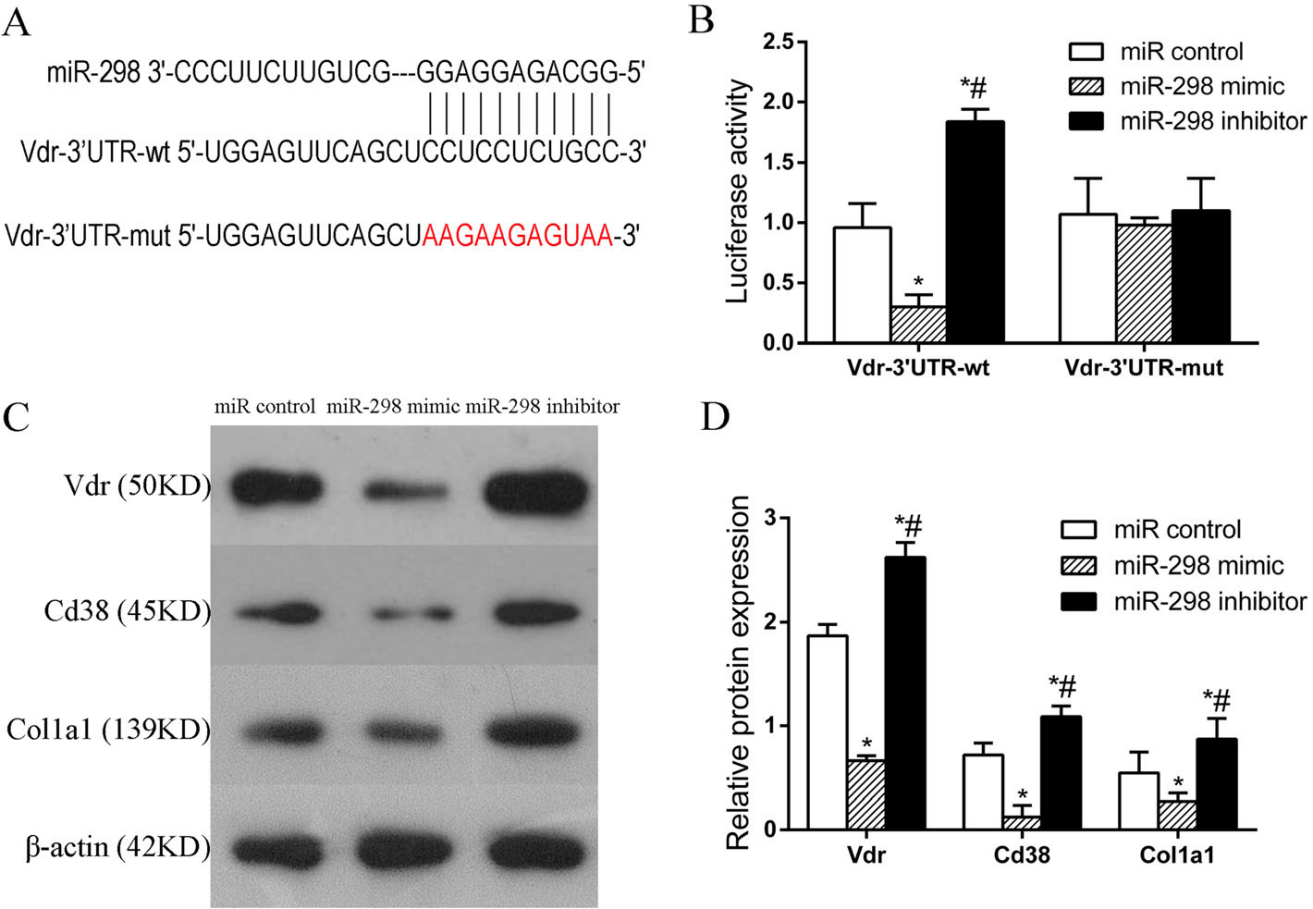
miR-298 directly targets Vdr during CN-induced MSC osteoblast differentiation. **a** Predicted miR-298 target sequence in the 3’UTR of Vdr. **b** Luciferase reporter assay of MSCs co-transfected with wt or mut Vdr plasmids and miR control, miR-298 mimic or miR-298 inhibitor, which showed that overexpression of miR-298 remarkably inhibited the luciferase activity of the wild-type Vdr 3’UTR (Vdr-3’UTR-wt) construct but not that of the mutated Vdr 3’UTR (Vdr-3’UTR-mut) construct. **c** and **d** Western blot detection of Vdr, Cd38, and Col1a1 protein expression in MSCs transfected with miR-298 mimic or miR-298 inhibitor. miR control group: MSCs transfected with miR control; miR-298 mimic group: MSCs transfected with miR-298 mimic; miR-298 inhibitor group: MSCs transfected with miR-298 inhibitor. *P < 0.05 vs. the miR control group. #*P* < 0.05 vs. the miR-298 mimic group

To gain insight into the molecular mechanisms by which miR-298 regulates CN-induced MSC osteogenic differentiation, we found that the osteogenic-specific gene vitamin D receptor (Vdr) has a miR-298 binding site in its 3’UTR. To test whether miR-298 directly targets Vdr, we constructed luciferase reporters that had either a wild-type 3’UTR (Vdr-3’UTR-wt) or a 3’UTR containing mutant (Vdr-3’UTR-mut) sequences of the miR-298 binding site. Overexpression of miR-298 remarkably inhibited the luciferase activity of Vdr-3’UTR-wt but not that of Vdr-3’UTR-mut (Fig. 5a & b). These findings indicated that miR-298 may directly regulate Vdr expression. Western blot analysis confirmed that miR-298 overexpression markedly suppressed the expression of Vdr, Cd38 and Col1a1 at the protein level (Fig. 5c & d). These results indicate that Vdr is a miR-298 target gene.

### miR-298 negatively regulates the expression of osteogenesis-related factors

**Fig. 6:**
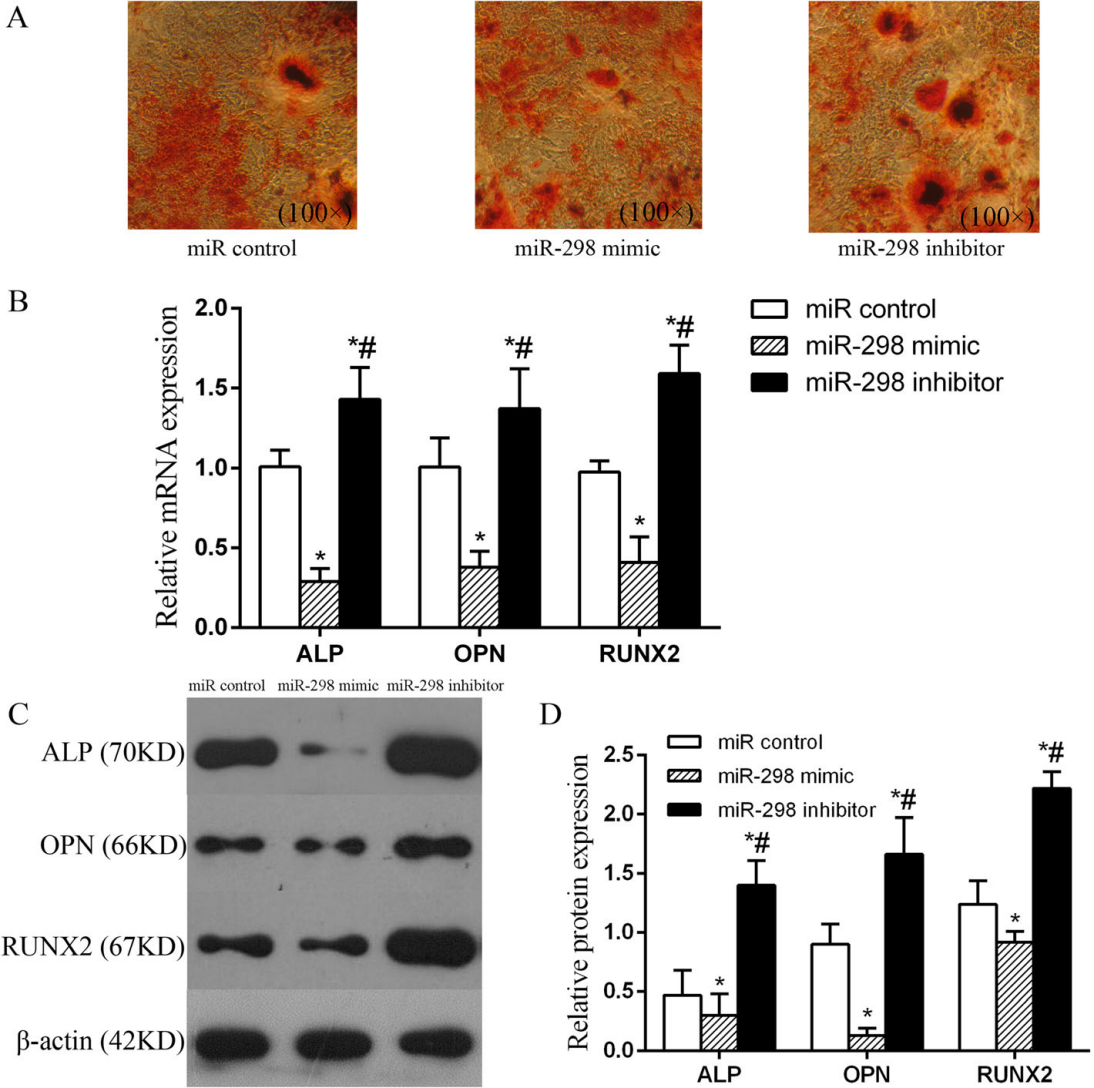
miR-298 negatively regulated osteogenesis-related factors during CN-induced MSC osteoblast differentiation. **a** AR-S showed that more calcium mineral deposition was found in the miR-298 inhibitor group than in the miR control and miR-298 mimic groups; in other words, miR-298 suppressed MSC osteogenic differentiation. **b** qRT-PCR detection of the mRNA expression of osteogenic-related factors in MSCs transfected with miR-298 mimic or miR-298 inhibitor showing that the mRNA levels of the osteogenic-related factors were decreased in the miR-298 mimic group compared with those in the miR control group, whereas these levels were enhanced in the miR-298 inhibitor group. **c** and **d** Western blot detection of the protein expression of osteogenic-related factors in MSCs transfected with miR-298 mimic or miR-298 inhibitor; the results were similar to the qRT-PCR results. miR control group: MSCs transfected with miR control; miR-298 mimic group: MSCs transfected with miR-298 mimic; miR-298 inhibitor group: MSCs transfected with miR-298 inhibitor. *P < 0.05 vs. the miR control group. #*P* < 0.05 vs. the miR-298 mimic group

To evaluate the role of miR-298 in the osteoblast differentiation of MSCs, we transfected MSCs with a miR control, mimic miR-298 mimic or miR-298 inhibitor construct. Then, the cells were cultured with CN for 7 days to induce osteogenic differentiation. The alizarin red staining (AR-S) assay showed that miR-298 significantly suppressed MSC osteogenic differentiation compared with that of cells transfected with the miR control. However, miR-298 inhibitor markedly promoted osteogenic differentiation (Fig. 6a). The mRNA and protein levels of osteogenesis-related factors, such as alkaline phosphatase (ALP), osteopontin (OPN) and runt-related transcription factor 2 (RUNX2), were analysed by qRT-PCR and western blot after miR-298 mimic transfection. The qRT-PCR results showed that the mRNA levels of the osteogenic-related factors were decreased in the miR-298 mimic group compared with those in the miR control group, whereas these levels were enhanced in the miR-298 inhibitor group (Fig. 6b). Western blot analysis also demonstrated changes in the protein levels of these osteogenesis-related factors, which were similar to the changes in the mRNA levels (Fig. 6c & d). These data suggest that miR-298 is the core miRNA functioning during the CN-induced osteoblast differentiation of MSCs.

### Vdr overexpression eliminated the inhibitory effect of miR-298 during CN-induced MSC osteoblast differentiation

**Fig. 7:**
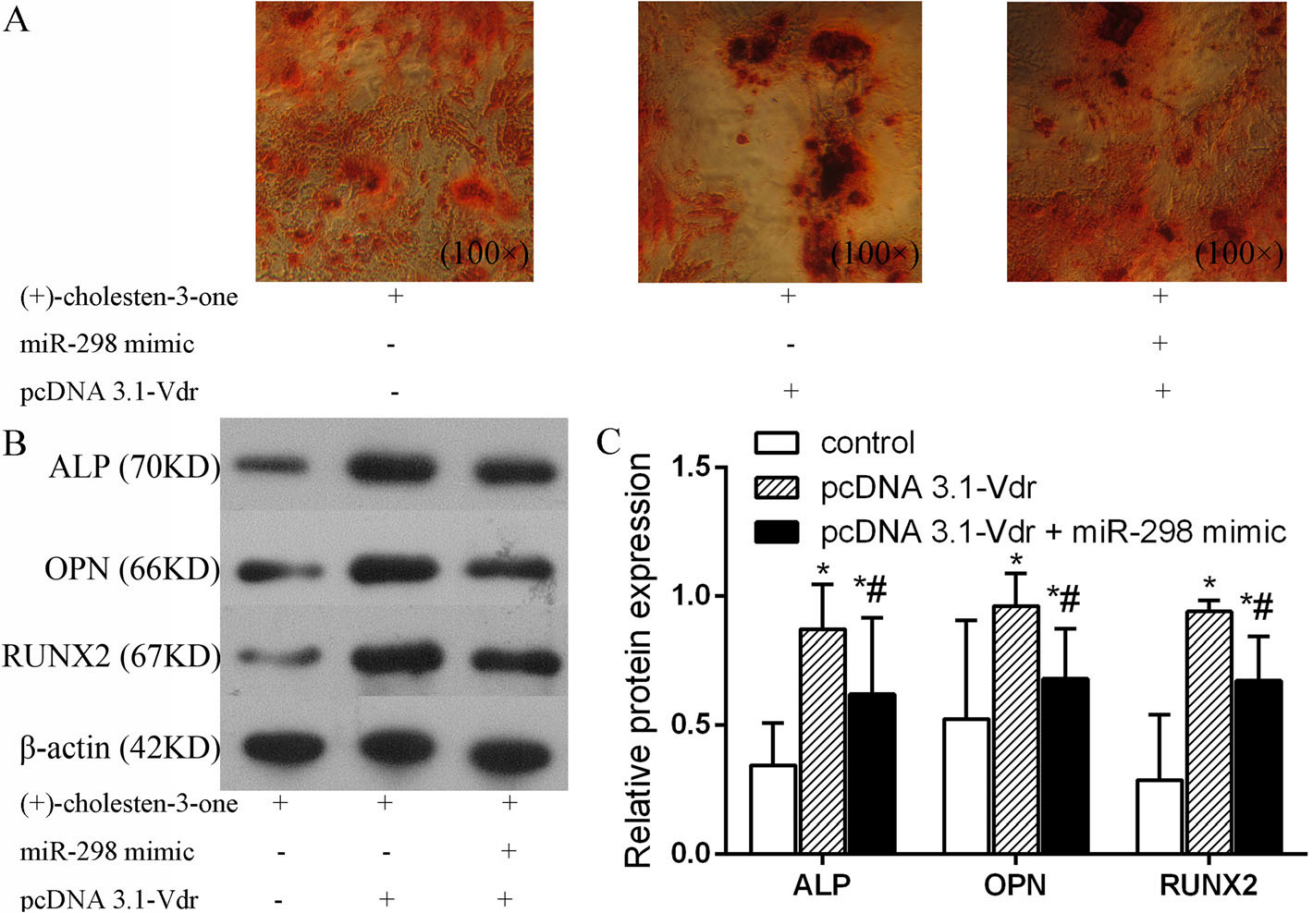
Vdr overexpression rescues the inhibitory effect of miR-298 during the CN-induced osteoblast differentiation of MSCs. **a** AR-S showed that more calcium mineral deposition was found in the CN + pcDNA 3.1-Vdr + miR-298 mimic group than in the CN group, while less calcium mineral deposition was found in the CN + pcDNA 3.1-Vdr group. Thus, pcDNA 3.1-Vdr rescued the inhibitory effect of miR-298 during the CN-induced osteoblast differentiation of MSCs. **b** & **c** Western blot detection of the protein expression of osteogenic-related factors in MSCs transfected with miR-298 mimic or pcDNA 3.1-Vdr; the results were similar to the AR-S results. Control group: MSCs transfected without pcDNA 3.1-Vdr or miR-298 mimic; pcDNA 3.1-Vdr group: MSCs transfected with pcDNA 3.1-Vdr; pcDNA 3.1-Vdr + miR-298 mimic group: MSCs co-transfected with pcDNA 3.1-Vdr and miR-298 mimic. *P < 0.05 vs. the CN group. #*P* < 0.05 vs. the miR-298 mimic group

To further understand the functional effect of miR-298 on osteogenesis-related factors, such as ALP, OPN and RUNX2, by regulating Vdr, we transfected MSCs with miR-298 mimic and pcDNA 3.1-Vdr under CN induction. The AR-S assay showed that more calcium mineral deposition was observed in the pcDNA 3.1-Vdr transfected group than in the untransfected control and co-transfected pcDNA 3.1-Vdr and miR-298 mimic groups (Fig. 7a). Furthermore, western blot analysis demonstrated that pcDNA 3.1-Vdr remarkably increased the expression of ALP, OPN and RUNX2. In addition, pcDNA 3.1-Vdr significantly reversed the inhibitory effects of miR-298 overexpression on osteogenic differentiation, restoring the expression of the osteogenic-related factors ALP, OPN and RUNX2 (Fig. 7b & c). These results indicate that Vdr overexpression abolished the inhibitory effect of miR-298 during the CN-induced osteoblast differentiation of MSCs.

## Discussion

The osteogenic differentiation of MSCs is precisely regulated by intrinsic molecular signals and the external environment [1]. Understanding the molecular mechanisms underlying MSC osteogenic differentiation is important for therapeutic purposes. Our previous reports [5, 6] indicated that CN has the potential to promote the osteogenic differentiation of MSCs. However, the role of epigenetic regulation in the CN-induced osteogenic differentiation of MSCs remains poorly understood. Recently, miRNAs have emerged as important regulators of the osteoblast differentiation of MSCs. Several studies [7, 23–25] have reported that miRNAs target the critical transcription factors involved in the osteoblast differentiation of MSCs. Growing reports have shown that a number of key signalling pathways are involved in the regulation of osteoblast differentiation, including the TGF-β/BMP signalling [22, 26], Wnt signalling [26, 27], Hedgehog signalling [28], Notch signalling [29, 30] and FGF signalling pathway [31]. Emerging evidence [32, 33] has revealed that lncRNAs may also play a vital role in regulating MSC osteogenic differentiation. For example, the regulation of miRNA-mRNA, lncRNA-mRNA, miRNA-lncRNA and miRNA-mRNA-lncRNA interactions has been reported in MSC osteogenic differentiation [21, 22]. However, the role of miRNA-mRNA-lncRNA interaction networks in the CN-induced osteogenic differentiation of MSCs remains largely unknown.

In this study, we outlined the miRNA-mRNA-lncRNA interaction network during CN-induced MSC osteogenic differentiation. Using miRNA and lncRNA microarray analysis, we identified 77 miRNAs (36 upregulated and 41 downregulated) and 295 differentially expressed lncRNAs, and 443 mRNAs were predicted based on the miRNA and lncRNA negative correlations. We then performed a dynamic analysis of the miRNA-mRNA-lncRNA interaction network during CN-induced MSC osteogenic differentiation, which suggested that downregulated miRNAs, upregulated lncRNAs and mRNAs were predominant.

Further bioinformatic analysis was performed to screen the key genes controlling CN-induced MSC osteogenic differentiation. First, GO cluster analysis identified 113 upregulated biological processes and 61 downregulated biological processes. Among the upregulated biological processes, the Vdr signalling pathway, response to mechanical stimulus, positive regulation of cell migration, positive regulation of cell proliferation, and collagen fibril organization processes, among others, may be related to CN-induced MSC osteogenic differentiation. These results were also consistent with previous reports [34–39]. Among the downregulated biological processes, the positive regulation of protein localization to early endosomes, cell proliferation, positive regulation of cell adhesion, and response to hypoxia processes may be related to CN-induced MSC osteogenic differentiation; these results were also consistent with those previously reported [40–44]. KEGG analysis was then used to identify 26 signalling pathways significantly related to CN-induced MSC osteogenic differentiation, including the axon guidance, TNF signalling, PI3K-Akt signalling, calcium signalling, mineral absorption, HIF-1 signalling, regulation of actin cytoskeleton, and focal adhesion pathways. Kim et al. used a fractionated secretomic approach and identified the axon guidance molecule SLIT3 as a clastokine that stimulates osteoblast migration and proliferation by activating β-catenin and confirmed that SLIT3 inhibits bone resorption by suppressing osteoclast differentiation in an autocrine manner [45]. Bai et al. found that TNF-α inhibits osteoblast differentiation mainly by activating the nuclear factor (NF)-κB signalling pathway [46]. Using in vivo and in vitro analyses, Xi et al. indicated that the PI3K/Akt cell signalling pathway is involved in osteoporosis inhibition by promoting osteoblast proliferation, differentiation and bone formation [47]. The calcium signalling pathway and mineral absorption have also been reported previously [48, 49].

Bioinformatic analysis of the miRNA-mRNA-lncRNA interaction network identified 16 core genes, including 6 mRNAs (Vdr, Mgp, Fabp3, Fst, Cd38, and Col1a1), 5 miRNAs (miR-483, miR-298, miR-361, miR-92b and miR-155), and 5 lncRNAs (NR_046246.1, NR_046239.1, XR_086062.1, XR_145872.1 and XR_146737.1). Vdr, a ligand-activated transcription factor, has been reported to be a core gene during MSC osteogenic differentiation [5, 6, 50], and matrix gla protein (Mgp) has been recognized as a potent calcification inhibitor and regulator of bone morphogenetic protein-2 (BMP-2) [51]. Wang et al. [52] indicated that overexpression of fatty acid binding protein 3 (Fabp3) inhibited MSC growth and proliferation via negatively regulating the cell cycle and MSC growth factors and enhancing cell survival under hypoxic or ischaemic conditions, which indicated that the Fabp3 gene potentially promotes MSC osteogenic differentiation. Gajos-Michniewicz et al. indicated that overexpression of follistatin (Fst) negatively influences bone metabolism and induces a significant decrease in biomechanical strength variables in FST-overexpressing mice compared to those in control mice. Overexpression of FST leads to decreased skeletal quality, thus increasing susceptibility to bone fractures [53], and Cd38 expression may increase the intracellular calcium concentration [54]. Col1a1, a marker of osteogenic differentiation, shows higher expression levels during the osteoblast differentiation of bone marrow stromal cells throughout osteogenesis [55]. The reliabilities of the sub-core CNC networks and online databases were verified via qRT-PCR and western blot.

A high degree of enrichment means that enrichment plays an important role in regulating CN-induced MSC osteogenic differentiation. Among the core miRNAs, mRNAs and lncRNAs, miR298 was the most significantly downregulated and associated with 6 enriched genes, while Vdr was associated with 3 enriched genes. Subsequent bioinformatics analysis revealed that miR-298 interacted closely with Vdr. Coincidentally, our previous study showed that Vdr positively regulates osteoblast differentiation induced by CN [5, 6]. We then further examined the function of miR-298 in MSC osteogenic differentiation using transfection methods and found that miR-298 significantly inhibited MSC osteogenic differentiation. miR-298 upregulation decreased osteoblast differentiation by inhibiting Vdr in MSCs. Following osteoblast differentiation induced by CN, miR-298 was significantly downregulated, and Vdr was no longer inhibited by miR-298, thereby facilitating the escape of MSCs from the quiescent state into osteogenic differentiation. However, how Vdr and miR-298 precisely regulate the osteogenic differentiation of MSCs requires further study. Nonetheless, the above results provide sufficient evidence that Vdr is a predicted miR-298 target gene. Bioinformatics analysis and a luciferase reporter assay verified that miR-298 inhibited the expression of Vdr via directly binding the 3’UTR region of its mRNA. Overexpression of Vdr markedly reversed the inhibitory effects of miR-298, indicating that Vdr was the effector of miR-298 during MSC osteogenic differentiation. Taken together, these results indicate that miR-298 may be a key factor in inhibiting MSC osteogenic differentiation and that Vdr is a miR-298 target gene.

Our data also showed that the expression levels of NR_046246.1, NR_046239.1, XR_086062.1, XR_145872.1 and XR_146737.1 were increased, while those of miR-483, miR-298, miR-361, miR-92b and miR-155 were decreased during CN-induced MSC osteogenic differentiation. Because miR-298 performed outstandingly in both the microarray and bioinformatics analyses, we treated it as the centre of our network. We used miRDE (http://www.mirdb.org), starBasehttp://starbase.sysu.edu.cn, and Segal Lab of Computational Biology (https://genie.weizmann.ac.il/pubs/mir07/mir07_prediction.html) to predict the binding sites between miR-298 and the other core lncRNAs, but no unequivocal relationships were observed. While none of the core lncRNAs have been reported previously, they may act as “sponges” to other miRNAs that we did not evaluate in this study, and we will conduct further examinations to verify their roles in the network. The lncRNAs KCNQ1OT1 [56], MALAT [57], XR_111050 [22], etc. reportedly promote osteogenic differentiation, while the lncRNAs HOTAIR [58], DANCR [59], TSIX [60], etc. reportedly inhibit osteogenic differentiation. miR-483-5p is overexpressed in osteoporotic patients and targets the downregulation of IGF2 expression [61], while miR-92b promotes cell proliferation and invasion in osteosarcoma by targeting DKK3 [62], and miR-155 inhibits osteoblast differentiation by downregulating the translation of SMAD5 in mouse pre-osteoblasts [63].

## Conclusions

We dynamically analysed the interaction network of coding and noncoding transcripts in CN-induced MSC osteogenic differentiation. Further bioinformatic analyses and in vitro experiments confirmed that miR-298 is required for CN-induced MSC osteogenic differentiation. Our study laid the foundation for further understanding the exact mechanism underlying the miRNA-mRNA-lncRNA interaction network regulating MSC osteogenic differentiation. In addition, the miRNAs and lncRNAs that are differentially expressed during MSC osteogenic differentiation may be potential targets for bone tissue engineering.

## Methods

### Isolation, culture and CN-induced MSC osteoblast differentiation

Sprague dawley rats were used in this study and euthanised by cervical dislocation. MSCs were isolated and cultured as described in our previous reports [5, 6] (Supplement 1). The initial culture medium was then replaced with fresh medium supplemented with 30 μg/mL CN, 10% FBS, and 1% penicillin-streptomycin to induce MSC osteoblast differentiation for 7 days and refreshed every 3 days thereafter.

### miRNA and lncRNA microarray analysis

After CN-induced osteoblast differentiation, MSCs were subjected to miRNA and lncRNA microarray assays performed by RiboBio Co., Ltd. (Guangzhou, China). The method was the same as that used in our previous report [6] (Supplement 2).

### Quantitative real-time polymerase chain reaction (qRT-PCR)

qRT-PCR was used to verify the expression of miRNAs, lncRNAs and mRNAs and to detect the expression of osteogenic differentiation markers as described in our previous report [6] (Supplement 3). Primer sequences are listed in Additional file 8.

### Candidate mRNA prediction

Candidate mRNAs targeted by the differentially expressed miRNAs identified in this study were predicted by Genminix Informatics Ltd., Co. (Shanghai, China) using miRanda (http://miranda.org.uk/). The miRanda method is based on dynamic programming (Smith-Waterman (SW) algorithm [64]) and the computation of free energy. When the sequence alignment score value (miRNA and 3’-UTR) and free energy are greater than the corresponding pre-defined thresholds, the gene for which the 3’-UTR is a sub-sequence is considered the target gene of the miRNA. During this process, the concrete alignment is obtained.

During alignment, every pair of bases has a score value (may be negative), and the ultimate score is the sum of all the values. The matched score value is greater than the mismatched score value, and the term wobble is used when the mismatched pair, T and G, has a greater score value than the other mismatched pairs. Insertions or deletions may occur during alignment, which are represented by “-”. Every gap has a penalty (negative sore value), and open gaps have a smaller penalty than extensive gaps. miRanda defines seeds, but gaps at seeds are not allowed, and positive score values and penalty score values occur several times more often in seeds than in non-seed areas.

### Construction of the CNC network

The CNC network was built according to the normalized signal intensity of miRNAs, lncRNAs and mRNAs. We calculated the Pearson correlation coefficient between two genes, and only the strong correlations (> 0.9) were selected to construct the CNC network, which was drawn with Cytoscape 3.0. Construction and visualization of the network were performed by Genminix Informatics Ltd., Co. (Shanghai, China).

### GO and KEGG analyses

GO and KEGG analyses were performed to identify the biological processes, molecular functions, cellular components and pathways as previously described [21] (Supplement 4).

### Core CNC networks

The core CNC network were then constructed based on the core genes(29 mRNAs), which were obtained from the same genes from GO and KEGG analyses. Two online databases (https://geneticassociationdb.nih.gov/, https://www.ncbi.nlm.nih.gov/gene/) were used to identify the osteogenic differentiation-related genes in the core CNC networks. The core genes identified from the core CNC networks and online databases were verified by qRT-PCR and western blotting [22].

### Bioinformatic analysis

miRanda (http://www.microrna.org), PicTar (http://pictar.mdc-berlin.de) and targetScan (http://www.targetscan.org) were used to analyse potential miR-298 binding sites on the Vdr 3’-UTR. The consistency of the analyses by these three websites suggested that the results were reliable, and the results were further verified using in vitro experiments.

### Luciferase reporter assays and MSC transfection with miR-298 mimic, miR-298 inhibitor and pcDNA 3.1-Vdr

Luciferase reporter assays were performed to identify whether miR-298 directly targets Vdr expression, as described in our previous study [6] (Supplement 5). MSCs transfected with miR-298 mimic, miR-298 inhibitor, or Vdr-overexpressing vector(pcDNA 3.1-Vdr, a Vdr recombinant vectors without 3’UTR) were subsequently subjected to AR-S and western blot analyses to clarify the role of miR-298 in MSC osteogenic differentiation as described in our previous report [6] (Supplement 6).

### Calcium mineral deposition

After transfection with a miR-298 mimic, miR-298 inhibitor or Vdr-overexpressing vector, MSCs continued to differentiate into osteoblast via CN for 14 days. AR-S was used to detect the osteogenic differentiation ability mediated by miR-298 and pcDNA 3.1-Vdr as described in our previous report [6] (Supplement 7).

### Western blot

Osteogenic-specific factors, such as ALP, OPN and RUNX2 were detected by western blot after the transfection of MSCs with miR-298 mimic, inhibitor and pcDNA 3.1-Vdr as described in our previous report [6] (Supplement 8).

### Statistical analysis

Statistical analyses were performed using SPSS 19.0 (SPSS Inc., Chicago, IL, USA). Statistical analyses included the t-test, Fisher’s exact test, χ^2^ test, one-way analysis of variance (ANOVA), least significant difference (LSD) and Pearson correlation based on the data type. *P* < 0.05 was considered statistically significant.

## Additional files

Additional file 1: miRNA and lncRNA microarray of MSC osteoblast differentiation induced by CN. (XLS 21 kb)

Additional file 2: Target gene prediction via mRBase targets based on the miRNA and lncRNA microarray. (XLS 155 kb)

Additional file 3: Relationship between miRNAs, mRNAs and lncRNAs according to the negative regulation. (XLSX 144 kb)

Additional file 4: GO analysis of target genes obtained from CNC networks. (XLSX 52 kb)

Additional file 5: KEGG pathway analysis of target genes obtained from CNC networks. (XLSX 31 kb)

Additional file 6: The core CNC networks were analysed based on the 29 mRNAs obtained from GO and KEGG pathway analyses. (XLSX 13 kb)

Additional file 7: Core CNC network analyses based on the online databases. (XLSX 42 kb)

Additional file 8: Primer sequences used for qRT-PCR. (XLSX 9 kb)

Additional file 9: Supplementary Materials. (DOC 37 kb)

## Abbreviations

CN: (+)-cholesten-3-one
MSCs: Mesenchymal stem cells
miRNAs: MicroRNAs
lncRNAs: Long-chain noncoding RNAs
CNC: Coding-noncoding gene co-expression
GO: Gene ontology
KEGG: Kyoto Encyclopedia of Genes and Genomes
Vdr: Vitamin D receptor
AR-S: Alizarin red staining
ALP: Alkaline phosphatase
OPN: Osteopontin
RUNX2: Runt-related transcription factor 2
Mgp: Matrix gla protein
BMP-2: Bone morphogenetic protein-2
Fabp3: Fatty acid binding protein 3
qRT-PCR: Quantitative real-time polymerase chain reaction
ANOVA: One-way analysis of variance
LSD: Least significant difference
