# Supplementary material for: Profiling the miRNA-mRNA-lncRNA interaction network in MSC osteoblast differentiation induced by (+)-cholesten-3-one
Source: BMC Genomics. 2018 Oct 29;19:783. doi: 10.1186/s12864-018-5155-2 (PMC6206902; doi:10.1186/s12864-018-5155-2)
Supplement: Supplementary file 9 — Supplementary Materials. (DOC 37 kb) [file 12864_2018_5155_MOESM9_ESM.doc]

**Supplementary Materials**

**1. Isolation, Culture of MSCs**

A total of 10 male specific pathogen free Sprague-Dawley rats, aged 4 weeks (180-200 g), were acquired from the Animal Centre of Guangzhou University of Chinese Medicine (Guangzhou, China). All animals received humane care in accordance with the guidelines set out by the Care of Experimental Animals Committee of Guangzhou University of Chinese Medicine. All rats were anaesthetized by chloral hydrate (330 mg/kg) and sacrificed by cervical dislocation, then the femur and tibia were harvested from rats sterilely, in order to collect fresh marrow. The marrow was mixed with complete medium (low glucose DMEM supplemented with 10% FBS) and gradient centrifuged at 900 x g for 30 min at room temperature with Percoll at a density of 1.073 g•ml^-1^. Cells of the appropriate density were collected, washed with PBS three times, manually counted using a light microscope and cultured at a density of 1x10^6^ cm^-2^ on dishes supplemented with complete medium, in a humidified atmosphere at 37 ℃ and 5% CO_2_, using an incubator. The medium was refreshed and the suspension cells were removed every three days. When cells were 80-90% confluent, MSCs were digested using 0.25% trypsin in order to promote separation. Cells were subsequently passaged at a density of 1x10^4^ cm^-2^ onto dishes. MSCs that had been passaged three times were used for subsequent experiments.

**2. miRNA and lncRNA microarray analysis**

The MSCs induced by CN for 7 days was taken to microarray assay which was performed at Guangzhou RiboBio Co., Ltd. The experiment contains 4 steps, like pre-hybridization, hybridization, hybridization washing and imaging. CustomArray^TM^ microarray was assembled with hybridization cap and clips. Firstly, it’s pre-hybridization: The hybridization chambers were filled with nuclease-free water to incubate at 65℃ for 10 min, and then bring to room temperature. The pre-hybridization solutions were following filled into the chambers after the water aspirating out of the hybridization chambers, and also kept on incubating at 37℃ for 60 min with gentle rotation in the hybridization oven. Secondly, the hybridization was performed: The hybridization solution was prepared like the following steps, total RNA from the hippocampus in both groups were extracted with trizol method and labeled with cy3 as fluorescence labeling by ULS^TM^(UNIVERSAL LINKAGE SYSTEM) notation, and then the solution was denatured at 95℃ for 3 min and cooled for 20 seconds on ice to prepare the hybridization steps. The hybridization chambers were filled with the hybridization solution as the followed that the pre-hybridization solution was taken out and mixed gently to incubate at 37 ℃ for 16 hours. Finally, the microarray was rinsed to decrease specific hybridization background, and then covered with the imaging solution and loaded into the GenePix 4000B Microarray Scanner to scan.

Two-class differentiation were used to normalize data from each array, which is applicable to analyses of small samples. Random variance model (RVM) t-test was applied to filter differentially expressed miRNAs and lncRNAs between 2 groups. P values (<0.05) and Fold change(>2) were used to screen miRNAs and lncRNAs respectively with significantly different expression. Hierarchical clustering of miRNAs and lncRNAs was using person correlation, which was performed using the Cluster 3.0 software and visualized with R Project (version 3.5.1).

**3. Quantifitative real-time polymerase chain reaction(qRT-PCR)**

Total RNA of MSCs was also extracted using TRIzol Reagent. qRT-PCR reactions were performed using the SYBR Green PCR, included 2-minute incubation at 50℃, then 95℃ for 10 minutes; this was followed by a 2-step PCR program, as follows: 95℃ for15 seconds and 60℃ for 60 seconds for 40 cycles.Relative mRNA levels were calculated using the 2^−ΔΔCt^ method. The ΔCts were obtained from Ct normalized with β-actin. All procedures were repeated three times. Pearson’s correlation coefficient was further calculated for each gene using the normalized data to quantify the consistency between microarray experiments and qRT-PCR (p<0.05 and R>0.9).

**4. GO and KEGG Analyses**

Gene Ontology database (http://www.geneontology.org) was used to perform GO analysis on the predicted target mRNAs. After the analyses for significance and false discovery rate (FDR), GO terms were selected from the significantly enriched gene sets. Pathway analysis was used to identify significant pathways for the predicted target mRNAs according to the Kyoto Encyclopedia of Genes and Genomes. The significant pathways were selected by Fisher’s exact and chi-square tests.

**5. Luciferase reporter assay**

Luciferase reporter assay was performed using the Firefly Luciferase Reporter Gene Assay Kit (Beyotime Biotechnology), according to the manufacturer's instructions. Briefly, wild-type and mutant Vdr (without miR-298 binding sites) plasmids were co-transfected with 100 ng of pre-miR-298 or pre-miR-ctrl into MSCs using Lipofectamine 2000 (Invitrogen). pRL-TK Vector that express Renilla luciferase served as an internal reference. Luciferase activity was measured using a dual luciferase assay kit (Promega, Madison, WI, USA) according to the manufacturer's instructions.

**6. Transfection of MSCs with miR-298 mimics, inhibitor or pcDNA 3.1-Vdr**

When cells were 80-90% confluent, the cells were passaged using trypsin digestion, then MSCs were harvested and plated into 6 well cell culture cluster at a density of 2×10^4^ cells per cm^2^ and transfected with 100nM miR-298 mimics, inhibitor, or Vdr overexperssing vector(pcDNA 3.1-Vdr, a Vdr recombinant vectors without 3’UTR) for 6 h according to the protocol of Lipofectamine 2000. And then MSCs were subsequently to conduct alizarin-red staining (AR-S), qRT-PCR and western blot.

**7. Calcium mineral deposition**

MSCs were co-cultured with CN for 14 days. The level of calcium mineral deposition was revealed using AR-S. Following for 14 days, MSCs were fixed with 70% ethanol, treated with 40 mM alizarin-red solutionfor 10 min at pH 4.2. Subsequently, MSCs were treated with 10% cetylpyridinium chloride in 10 mM sodium phosphate for 15 min and then washed with PBS for 15 min. Calcium mineral deposition was observed at three different views(100×) to compare the drgree of osteogenic differentiation between different groups.

**8. Western blot**

The protein extracts from MSCs were separated by 10% SDS-PAGE and the blot was probed with primary antibodies (Abcam, Cambridge, UK). Proteins were transferred onto a PVDF membrane according to the manufacturer’s protocol and the membrane was blocked with 5% non-fat milk in PBST solution for 4℃ overnight, followed by a second antibody labeled with horseradish-peroxidase at room temperature for 1 hour, and then washed with 0.1% TBST. the protein expression was detected using a SuperSignal West Femto Maximum Sensitivity Substrate Kit(Roche Applied Science, Mannheim, Germany). The relative value of the target protein was calculated by comparing with the corresponding internal reference.
